# Wearable-Derived Axis-Specific Motor Signatures of ADHD Symptoms in Children and Adolescents

**DOI:** 10.3390/bios16060323

**Published:** 2026-06-02

**Authors:** Siyu Zhang, Jingsong Liu, Shoujiang Wu

**Affiliations:** 1 Department of Physical Education, Bozhou University, Bozhou 236800, China; 2 Department of Sport Science, College of Natural Sciences, Jeonbuk National University, Jeonju 54896, Republic of Korea

**Keywords:** ADHD, wearable accelerometry, motor behavior, movement fragmentation, adolescents, digital phenotyping

## Abstract

ADHD is typically assessed through reports from parents, teachers, or clinicians, but these reports may not fully capture how motor behavior is organized at the signal level. This cross-sectional study examined whether X-axis acceleration features derived from a wearable device could provide preliminary evidence of ADHD symptom-related motor-pattern differences in children and adolescents. Primary school children aged 6–13 years wore an Apple Watch Series 7, and X-axis accelerometer signals were used to extract features reflecting waveform distribution, zero-crossing rate, micro-motion, and local movement fragmentation. ADHD symptoms and broader emotional and behavioral difficulties were assessed using the SNAP-IV and SDQ. The results showed that the X-axis zero-crossing rate was the most robust feature differentiating the High ADHD and Low ADHD groups across the T-task and F-task recordings. X-axis zero-crossing rate reached statistical significance (*p* = 0.029), indicating more frequent short-interval switching between positive and negative acceleration directions in children with higher ADHD symptom levels. This finding suggests that directional switching or local movement fragmentation in X-axis acceleration may be a sensitive movement characteristic associated with ADHD symptoms. In addition, X-axis skewness showed a consistent directional tendency, with higher values in the High ADHD group; this effect was marginal in the T-task recording (*p* = 0.065), suggesting a possible tendency toward waveform asymmetry or distributional imbalance during longer movement recording. Overall, these findings provide preliminary evidence that ADHD symptom-related motor differences may be reflected in the organization of X-axis acceleration signals, particularly in directional switching indexed by zero-crossing rate and, to a lesser extent, waveform asymmetry indexed by skewness. Given the cross-sectional design, symptom-based grouping, modest sample size, and incomplete recording-context control, these results should be interpreted cautiously and require confirmation in larger, diagnostically characterized samples with standardized wearable-recording protocols.

## 1. Introduction

Digital assessment has become an increasingly important direction in pediatric attention-deficit/hyperactivity disorder (ADHD) research because conventional symptom evaluation still relies heavily on subjective behavioral reports and brief clinical observation [1,2]. Recent advances in wearable sensing and digital phenotyping have enabled continuous and ecologically grounded monitoring of movement dynamics, behavioral dysregulation, and motor variability in real-world settings. Compared with questionnaire-based evaluation alone, wearable-derived behavioral monitoring may provide a more objective representation of how ADHD-related characteristics emerge during everyday activity [1,2]. More broadly, wearable technologies are being used with increasing frequency across child and adolescent mental health, reflecting a shift from single-session testing toward repeated and ecologically grounded measurement [2,3]. This trend is especially relevant in ADHD, where symptom expression often varies across settings and over time, and where behavioral dysregulation may be more visible in naturalistic observation than in brief clinic-based encounters [1,3,4].

Among available digital tools, wrist-worn motion sensing is particularly attractive because it allows continuous and relatively low-burden data collection in everyday or semi-structured settings. Several studies have shown that smartwatch- or actigraphy-derived signals can detect meaningful behavioral differences in children and adolescents with ADHD. In a school-based study, Lin et al. reported higher movement variability and more frequent directional switching in children with ADHD than in controls, particularly in variance- and zero-crossing-related features derived from smartwatch accelerometer and gyroscope data [5]. Lindhiem et al. further demonstrated that smartwatch-based sensing combined with machine learning could objectively quantify hyperactivity in school-age children [6]. In adolescents, Jiang et al. showed that wearable activity, heart rate, and sleep signals could support ADHD classification, and that performance improved when wearable measures were combined with questionnaire data [7]. Rahman et al. reported similar findings using Fitbit-derived features in adolescents [8]. Together, these studies indicate that wearable-derived movement signals contain clinically relevant information, but they also suggest that the informative content of the signal may lie in specific structural features of movement rather than in gross activity level alone [5,8].

A related line of work has long used actigraphy to examine sleep and activity in ADHD. Early studies showed that actigraphy could differentiate children with ADHD from controls and could also capture treatment-related changes in activity during sleep and wakefulness [9,10]. Subsequent work reported altered actigraphic sleep patterns, including longer sleep onset latency, lower sleep efficiency, and more movements during sleep in children with ADHD [11,12]. A meta-analysis by De Crescenzo et al. concluded that children with ADHD show higher mean daytime activity during structured sessions and a moderately altered sleep profile, highlighting the value of actigraphy as an objective tool for ambulatory monitoring [13,14,15]. More recent work has continued to refine this picture by examining sleep profiles over longer periods, ADHD presentations, and relations with broader mental health outcomes [16,17]. These findings reinforce the broader point that objective movement and sleep data are relevant to ADHD [18]. Building on this actigraphy-based evidence, digital wrist-worn devices provide a more granular approach to the assessment of ADHD-related motor behavior. Unlike traditional actigraphic outputs, which often summarize movement as global activity counts or sleep–wake parameters, contemporary smartwatches can capture triaxial acceleration signals at the waveform level. This makes it possible to examine axis-specific movement properties, waveform distribution, zero-crossing rate, micro-motion, and local fluctuation patterns. Such features may be particularly relevant for ADHD because symptom-related motor differences may not be limited to greater activity volume, but may also involve altered signal organization, including directional switching, waveform asymmetry, and short-interval movement fragmentation. Therefore, smartwatch-derived accelerometry offers a complementary digital framework for characterizing ADHD-related motor behavior beyond conventional activity summaries.

At the same time, several limitations in the current literature remain unresolved. First, many studies still emphasize aggregated activity counts, global intensity measures, or broad variability indices. Far less attention has been paid to waveform organization, local oscillatory density, short-interval reversals, axis-dependent asymmetry, and fragmentation of movement over time. Second, pediatric wearable studies vary considerably in sensor type, sampling rate, recording duration, body placement, feature extraction strategy, and validation design, making direct comparison difficult [1,4]. Third, recent reviews repeatedly note that the field no longer lacks candidate variables. The more pressing problem is the absence of stable, interpretable, and clinically meaningful feature definitions that generalize across settings [2,4]. In practical terms, the unresolved issue is not whether wearable devices can detect ADHD-related movement differences, but how those differences should be defined and interpreted [4].

This gap is particularly important when raw triaxial accelerometer waveforms are examined directly. Two children may show similar overall movement amplitude while differing substantially in fluctuation density, local reversals, waveform fragmentation, or axis-specific distributional structure. Such differences are difficult to capture through global activity summaries alone. Recent work using deep learning on actimetry has shown that time–frequency patterns in long activity records can distinguish ADHD from controls and may reveal age- and gender-related differences in signal organization [19]. Other studies have shown that the choice of recording interval and feature selection strategy can materially affect diagnostic performance in activity-based ADHD models, suggesting that how the signal is segmented and represented matters as much as the choice of classifier itself [20]. In addition, multimodal wearable studies integrating motion data with clinical profiles or other biosignals further support the view that objective ADHD assessment will likely depend on structured signal interpretation rather than on a single global activity marker [21,22,23].

Several movement features examined in the present study have also been explored in previous wearable and digital phenotyping research. Previous studies have reported that axis-specific movement properties and multidirectional movement variability are associated with altered motor regulation, postural instability, and hyperactivity-related behavioral dysregulation in children with ADHD and related neurodevelopmental conditions [24].

The features used in the present study were selected based on prior wearable-motion and ADHD-related research. Axis-specific accelerometer and gyroscope features have been previously used to quantify motor differences in children with ADHD. For example, Lin et al. [5] reported higher variance values across the x-, y-, and z-axes of both accelerometer and gyroscope recordings in children with ADHD, and also examined zero-crossing rate as an indicator of rapid back-and-forth movement patterns. Similarly, Ouyang et al. [24] showed that accelerometer variance along specific axes decreased after methylphenidate treatment and was partly correlated with reductions in SNAP hyperactivity scores. These findings support the use of axis-specific movement variability and short-interval reversal-related indicators as objective markers of hyperactivity and motor instability.

In addition, SNAP-IV has been widely used as a standardized behavioral rating scale for assessing core ADHD symptoms, including inattention, hyperactivity, and impulsivity Bussing et al. [25] The SDQ has also been widely applied in child and adolescent mental-health research, and its hyperactivity/inattention subscale provides a brief screening indicator of ADHD-related behavioral difficulties Goodman et al. [26] Previous studies have further used SDQ-based indicators to examine the associations between physical activity, ADHD symptoms, and broader psychosocial outcomes in children and adolescents van Egmond-Fröhlich et al. [27]; Ganjeh et al. [28]. Therefore, the inclusion of SNAP-IV and SDQ scores in the present study was not arbitrary; rather, these measures were selected based on established behavioral-assessment approaches and were used to complement the wearable-sensor-derived movement features.

These considerations make the present study necessary for three reasons. First, an objective description of motor behavior remains one of the most plausible ways to complement subjective ADHD ratings in children [1]. Second, it remains unclear whether the features that best separate symptom-defined groups are the same features that track symptom severity continuously across individuals [8]. Third, if wearable-derived markers are to become clinically interpretable, they must be described in behaviorally meaningful terms rather than as isolated statistical features. The present study addresses this need using triaxial accelerometer data collected from children’s Apple Watches. Rather than focusing only on overall movement level, we examined raw waveform organization and derived motion features across two recording formats with different sampling structures. Particular attention was given to axis-specific properties, local fluctuation patterns, and their relationships with SNAP-IV and SDQ scores. The aim was to determine whether ADHD-related motor differences are better characterized by a simple increase in movement output or by changes in the signal’s temporal and structural organization. By doing so, this study seeks to refine the description of wearable-derived motor phenotypes in pediatric ADHD and to provide a more interpretable framework for objective behavioral assessment.

Existing studies have shown that children with ADHD exhibit motor-function abnormalities. However, traditional assessment methods are often time-consuming and rely heavily on subjective judgment. Wearable sensors may provide objective and quantifiable motor indicators. Using a dual-duration recording design, the present study collected X-axis movement data from children with ADHD symptoms using a wrist-worn accelerometer and systematically examined between-group differences in motor features. The results showed that the X-axis zero-crossing rate was significantly higher in the high-symptom group during the short-duration recording (*p* = 0.029), whereas the X-axis skewness showed the largest effect size during the long-duration recording (*p* = 0.065). These findings suggest that X-axis movement alterations in children with ADHD symptoms may appear across different recording durations, with their expression partly modulated by recording length and task structure. This study provides empirical evidence for the potential use of wearable sensors in assisting the assessment of ADHD-related motor function.

## 2. Materials and Methods

### 2.1. Participants and Study Design

This study used a cross-sectional dataset derived from primary school children aged 6–13 years. The study protocol was approved by the relevant institutional ethics committee under approval number 202309801, and written informed consent was obtained from the legal guardians of all participants before data collection. In addition to wearable signals, demographic variables including age, sex, and body mass index (BMI) were recorded for each participant. The overall design was intended to support the examination of associations between objectively measured movement characteristics and child mental health outcomes, an approach increasingly advocated in pediatric digital mental health research.

### 2.2. Wearable Motion Data Acquisition

Physical activity data were collected using the Apple Watch Series 7. Triaxial accelerometer signals were sampled at 100 Hz and stored as raw acceleration time series. The dataset contained two accelerometer recording formats. T files represented longer continuous recordings sampled at approximately 30 Hz, whereas F files represented shorter 1 min recordings sampled at approximately 100 Hz. These two formats were analyzed separately because they differed in both recording duration and sampling frequency.

Triaxial acceleration data were recorded using an Apple Watch Series 7 smartwatch (Apple Inc., Cupertino, CA, USA), which was employed as a wrist-worn wearable sensing device. During each recording session, the device was securely attached to the participant’s wrist to capture acceleration signals along three orthogonal axes. The built-in accelerometer of the Apple Watch Series 7 was used to obtain movement-related acceleration data, which were subsequently processed for axis-specific feature extraction and movement-pattern characterization. Because the device is commercially available and minimally invasive, it was considered suitable for use with children and adolescents in naturalistic and task-based assessment settings. In this study, the Apple Watch was used primarily as a data-acquisition tool rather than as a diagnostic device.

These were subsequently processed into motion-derived features representing spatial and temporal movement characteristics. Wrist-worn inertial sensing has been shown to provide valid estimates of physical activity in school-aged children [24,29], and smartwatch-based motion features have previously been used to characterize ADHD-related movement patterns, including variability- and switching-related features, in both school-based and mobile sensing contexts [5,6,30].

### 2.3. Mental Health Assessment

Mental health characteristics were assessed using two parent-report instruments. The Strengths and Difficulties Questionnaire (SDQ) was used to evaluate broad emotional and behavioral attributes. The SDQ is a widely used 25-item screening measure for child and adolescent mental health and has demonstrated acceptable reliability and validity in both original and community validation studies [26,31]. ADHD-related symptoms were assessed using the Swanson, Nolan, and Pelham Version IV Scale (SNAP-IV), which measures core dimensions of attention-deficit/hyperactivity disorder. The SNAP-IV has shown satisfactory validity and reliability in child samples and is commonly used as a screening and symptom-rating instrument in ADHD research. Participants were not classified based on a formal clinical ADHD diagnosis. Instead, group assignment was derived from questionnaire-based symptom severity, primarily using SNAP-IV scores. Accordingly, the symptom-defined groups in this study represent relatively higher and lower levels of ADHD-related symptoms within the sample, rather than clinically confirmed ADHD and non-ADHD groups. Information on formal diagnostic procedures, diagnostic criteria, medication status, and systematic exclusion of psychiatric comorbidities was not available in the dataset. The SNAP-IV has previously demonstrated acceptable validity and reliability in pediatric ADHD research and is commonly used as a screening and symptom-rating instrument [32,33].

### 2.4. Analytical Applications of the Dataset

The resulting dataset was designed to support the analysis of relationships between wearable-derived movement patterns and children’s mental health outcomes. In addition to descriptive and correlational analyses, the dataset is suitable for multimodal modeling, including the joint use of accelerometer features, demographic variables, and questionnaire-based symptom indicators for ADHD-related classification and behavioral phenotyping. Recent work has shown that wearable signals can contribute to the identification of ADHD in adolescents and may provide useful complementary information when combined with symptom ratings [34].

### 2.5. Raw Data Source and File Structure

Wearable movement data were obtained from a wrist-worn triaxial inertial sensor and stored in CSV format. The raw dataset included multichannel motion recordings, comprising acceleration, gravity-corrected acceleration, gyroscope signals, Euler-angle-related variables, and step-related outputs. Across files, the dataset contained 55 columns of sensor-derived information. Symptom grouping was based on the mean SNAP-IV score, with participants classified into the high-symptom group when the mean score was ≥1.67 and into the low-symptom group otherwise.

### 2.6. Preprocessing Pipeline

A standardized preprocessing pipeline was applied before feature extraction. First, all signals were resampled from 100 Hz to 25 Hz using the scipy.signal.Resample function to harmonize the temporal resolution across analyses and reduce computational redundancy. Second, file delimiters were automatically detected during import because T files and F files used different separators. Third, timestamps were parsed from the original time column when available; if valid timestamps were missing, time was reconstructed on the basis of the nominal sampling frequency. Fourth, recordings were truncated to the predefined task intervals used for analysis. For the F condition, the first 4.5 min of valid data were retained, whereas for the T condition, the first 5 min were used in the primary window-based analysis. Finally, extreme values exceeding ±5 g were treated as invalid and replaced with missing values. All downstream computations were performed using NaN-robust summary functions to minimize bias from isolated artifacts.

### 2.7. Sliding-Window Strategies for the F and T Conditions

Because the F and T conditions differed substantially in duration and recording structure, they were analyzed using separate windowing strategies. For the F condition, which represented shorter activity-fragment recordings, three complementary strategies were used to capture both stable and transient movement patterns: (1) 30 s windows with a 15 s step; (2) 60 s windows with a 30 s step; and (3) shorter fixed segmentation schemes, including 10 equal 6 s segments and 9 equal 30 s segments, to capture local temporal variability and brief movement bursts. For the T condition, which consisted of longer recordings, several layered strategies were used to examine both early-phase and longer-duration movement organization: (1) the first 5 min divided into 10 segments of 30 s; (2) the first 10 min divided into 10 segments of 60 s; (3) the first 30 min divided into 10 segments of 180 s; (4) rolling 60 s windows sampled across the full recording; and (5) coarse-grained comparisons across larger time blocks spanning the full recording.

This multi-window design was intended to reduce dependence on a single arbitrary segmentation choice and to identify features that remained stable across alternative temporal scales.

### 2.8. Time-Domain Features

A set of 11 time-domain features was extracted from each window for each device axis and, where applicable, for the composite acceleration magnitude. These features were selected to characterize baseline movement level, variability, fragmentation, and local oscillatory structure. Let the acceleration sequence for a given window be a(t) = {a1, a2, …, aN}, with sampling rate fs = 25 Hz and duration T = N/fs. The following features were calculated: mean, representing baseline movement level; standard deviation, calculated as sample standard deviation (ddof = 1), representing movement variability; root mean square (RMS), representing signal magnitude or movement intensity; skewness and kurtosis, characterizing distributional asymmetry and peakedness; zero-crossing rate (ZCR), defined as the number of sign changes per unit time and used as a proxy for directional switching and movement fragmentation; peak density, defined as the number of local maxima per unit time and used as an index of oscillatory density; micro-motion index (MMI), defined as the proportion of samples with absolute amplitude below a dynamic threshold based on within-window variability; and mean absolute first difference, used as an unscaled jerk-related descriptor reflecting abrupt local changes in acceleration.

Among these variables, ZCR, peak density, and MMI were treated as particularly relevant to fragmented and irregular movement organization.

### 2.9. Frequency-Domain Features

A further set of 11 frequency-domain features was extracted to characterize rhythmic organization and spectral complexity. Power spectral density (PSD) was estimated using the Welch method, with segment length Nseg = min(256, N), 50% overlap, and a Hanning window.

The extracted spectral features included spectral entropy, reflecting the distributional complexity of signal energy across frequencies; spectral centroid, representing the center of gravity of the power spectrum; spectral spread, indexing frequency dispersion around the centroid; dominant frequency, indicating the most prominent oscillatory component; band-specific RMS energy in the following ranges: VLF, 0–0.5 Hz; LF, 0.5–1.5 Hz; MF, 1.5–3.0 Hz; and HF, 3.0–12.0 Hz; and the LF/HF ratio, used as a summary descriptor of relative low- and high-frequency dominance. For band-limited features, signals were passed through third-order Butterworth filters and processed using filtfilt to ensure zero-phase filtering.

The mathematical definitions of the feature-extraction procedures are provided in the Supplementary Materials to support transparency and reproducibility.

### 2.10. Statistical Analysis Workflow

All statistical analyses were conducted independently for each windowing scheme. Because many feature distributions deviated from normality, group comparisons between the high- and low-symptom groups were performed using the two-tailed Mann–Whitney U test. To control for multiple testing across the primary feature set, a Bonferroni correction was applied as a conservative procedure, and a false discovery rate (FDR) adjustment was additionally examined as a sensitivity analysis.

For each group comparison, Cohen’s d was calculated to quantify effect size. To assess robustness across segmentation strategies, features were considered relatively stable when significant group differences were observed in three or more windows. In addition to categorical group comparisons, associations between movement features and continuous SNAP-IV scores were evaluated using Pearson correlation analysis.

Overall, the feature space comprised basic time-domain variables, frequency-domain variables, and composite descriptors across the three axes and derived measures, resulting in a high-dimensional window-level representation of movement organization. This framework was designed to characterize symptom-related differences in motor fragmentation, oscillatory structure, and axis- or orientation-related variation across both the F and T conditions.

## 3. Results

### 3.1. Sample Characteristics

Table 1 shows the Height and weight variables; each contained six missing cases, whereas BMI contained five. This minor discrepancy resulted from differences in data availability during the original preprocessing, with one participant retaining an available BMI record in the archived dataset. This discrepancy did not affect the subsequent statistical analyses.

### 3.2. ADHD-Related Motor Signatures in the F Group

**Figure 1:**
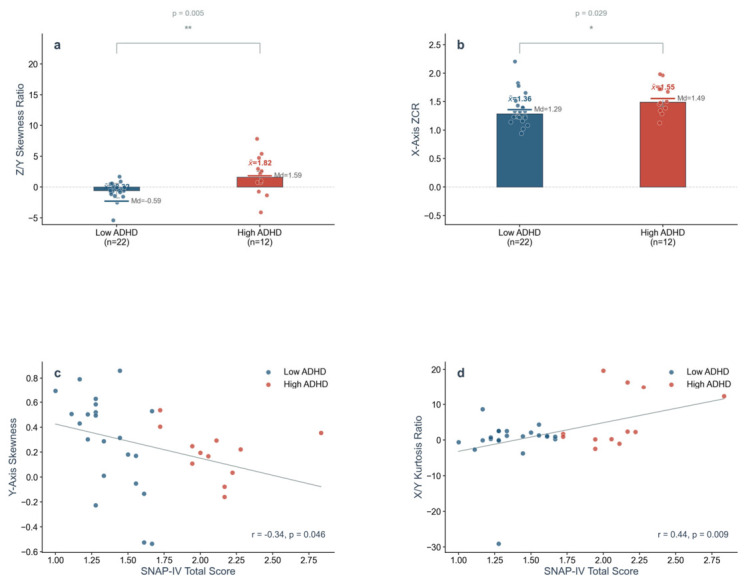
F Motor Fragmentation and Axial Asymmetry in ADHD. Note: * *p* < 0.05; ** *p* < 0.01.

Figure 1 shows that the Z/Y skewness ratio was significantly higher in the high-ADHD group than in the low-ADHD group (*p* = 0.005), indicating a stronger shift in directional asymmetry across motion axes. In addition, the X-axis zero-crossing rate was significantly elevated in the high-ADHD group (*p* = 0.029), consistent with more frequent directional changes and a more fragmented lateral movement pattern.

The unequal group sizes were due to the use of symptom-based grouping criteria rather than to artificial sample-balancing procedures. Participants were classified according to standardized ADHD-related thresholds, and therefore, the resulting distribution reflects the natural variability of symptom severity within the sample. In community and school-based populations, higher-symptom ADHD groups are typically smaller than lower-symptom groups. We intentionally retained the original distribution to preserve ecological validity and avoid introducing additional sampling bias through forced balancing.

The X/Y kurtosis ratio showed a significant positive correlation with SNAP-IV total score (Pearson r = 0.44, *p* = 0.009). These results indicate that increasing ADHD symptom burden was associated with progressive changes in the distributional structure of movement rather than with a uniform increase in overall motor output.

(a) shows the difference between the high-symptom and low-symptom groups in the Z/Y Skewness Ratio. This ratio reflects the relative asymmetry of acceleration waveform distributions between the vertical axis and the anteroposterior axis. A clear group difference in this ratio suggests that children in the high-symptom group may show an altered balance between vertical and forward–backward movement organization. In other words, the movement pattern of the high-symptom group was not simply characterized by greater or lower movement magnitude, but by a disproportionate relationship between axis-specific waveform asymmetries. This axial imbalance may indicate atypical coordination between vertical postural adjustments and anteroposterior movement regulation. From a motor-control perspective, such a pattern may be related to altered vestibular–proprioceptive integration or reduced efficiency in postural control, although this interpretation should be regarded as preliminary and requires further confirmation with larger samples and direct sensorimotor measures.
(b) presents the between-group difference in X-axis zero-crossing rate. ZCR refers to the number of times the acceleration signal crosses the zero baseline within a given time window, and it is commonly interpreted as an index of oscillatory switching, directional change, and rhythm fragmentation. The observed difference between the high-symptom and low-symptom groups indicates that children with higher ADHD symptoms may exhibit a more fragmented or irregular lateral movement pattern. Because the X-axis reflects left–right movement, this finding suggests that symptom-related motor differences may be particularly expressed in lateral movement regulation. A higher or more abnormal X-axis ZCR may reflect more frequent short-interval changes in movement direction, reduced rhythm stability, or less fluent motor control. This interpretation is consistent with the broader notion of motor disfluency in ADHD, in which movement is often described as less smooth, less stable, and more variable across time. Scatterplot showing the relationship between SNAP-IV total scores and Y-axis skewness. A significant negative correlation was observed, suggesting that greater ADHD symptom severity was associated with reduced asymmetry in lateral movement distribution.
(c) examines the linear association between Y-axis skewness and SNAP-IV total scores. The Pearson correlation did not reach statistical significance, indicating that Y-axis skewness was not a stable continuous predictor of ADHD symptom severity in the present sample. This means that although Y-axis waveform asymmetry may differ visually or descriptively between symptom groups, its linear relationship with SNAP-IV scores was not sufficiently strong to support a continuous symptom-severity interpretation. However, the scatterplot still suggests some degree of distributional separation between the high- and low-symptom groups, with the high-symptom group tending to show higher Y Skewness values. Therefore, Y Skewness may be more appropriately interpreted as a candidate group-discriminative feature rather than a robust continuous marker of SNAP-IV symptom severity.
(d) explores the relationship between the X/Y Kurtosis Ratio and SNAP-IV total scores. Kurtosis reflects the peakedness and tail structure of a signal distribution. A higher kurtosis value may indicate that the acceleration signal is concentrated around the mean while also containing intermittent extreme deviations, which can be interpreted as a possible sign of fragmented or burst-like movement patterns. The X/Y Kurtosis Ratio therefore captures the relative difference in distributional sharpness between the lateral and anteroposterior axes. Although the correlation with SNAP-IV total scores did not reach statistical significance, this feature may still provide information about axis-to-axis coordination rather than direct symptom severity. In this sense, the X/Y Kurtosis Ratio should not be interpreted as a reliable linear indicator of ADHD symptom scores in the present dataset. Instead, it may reflect subtle abnormalities in inter-axis movement organization, which could help characterize motor-pattern differences between symptom-defined groups when combined with the group-comparison results.

Dots represent individual participants. Bar plots are presented as mean ± standard error. Solid lines indicate linear regression fits. The SNAP-IV total score was used as the index of ADHD symptom severity.

In Group F, several accelerometer-derived motor features were compared between participants with higher and lower ADHD symptom levels. The Z/Y skewness ratio showed a significant between-group difference (*p* = 0.008) with a large effect size (d = 1.16), indicating a substantial shift in cross-axis asymmetry in the high-symptom group. Y-axis skewness was not statistically significant (*p* = 0.351), but its effect size was moderate (d = −0.56). In contrast, X-axis zero-crossing rate (*p* = 0.607, d = 0.15) and the X/Y kurtosis ratio (*p* = 0.758, d = 0.08) showed only negligible effects.

### 3.3. ADHD-Related Motor Signatures in the T Group

**Figure 2:**
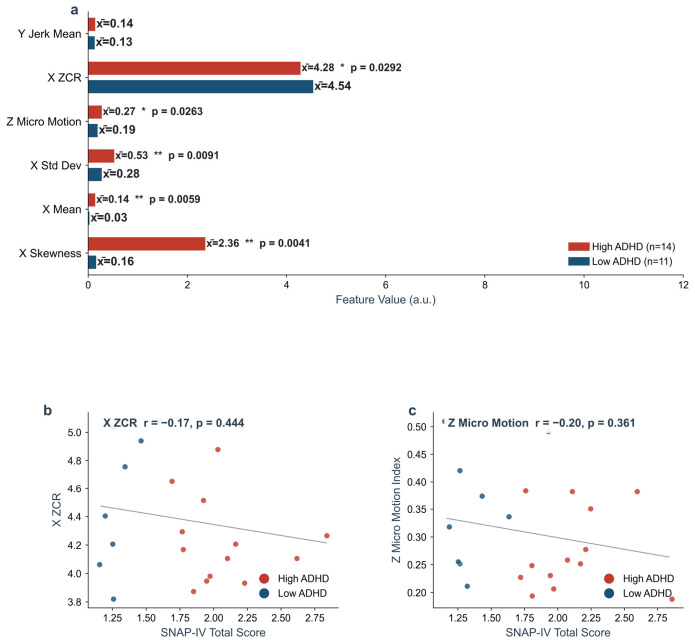
ADHD-Related Motor Features in Group T. Note: * *p* < 0.05; ** *p* < 0.01.

Figure 2. In Group T, several accelerometer-derived motor features differed significantly between children with high and low ADHD symptom levels. The strongest group difference was observed for X-axis skewness, which was markedly elevated in the high-ADHD group (*p* = 0.004). Significant between-group differences were also found for X-axis mean (*p* = 0.006), X-axis standard deviation (*p* = 0.009), X-axis zero-crossing rate (*p* = 0.029), and Z-axis micro-motion index (*p* = 0.026). By contrast, the Y-axis jerk mean did not differ significantly between groups. X-axis zero-crossing rate (d = −0.46) and X-axis mean (d = 0.47) both fell within the small-effect range, while X-axis skewness (d = −0.13), Y-axis jerk mean (d = −0.20), and Z-axis micro-motion (d = −0.16) showed negligible effects, suggesting limited feature-level separation between groups in the T condition.

(a) compares six movement-derived features between the high-symptom and low-symptom groups. The results show that several features differed between the two groups, particularly those extracted from the X-axis acceleration signal. Specifically, X Skewness, X Mean, X Std Dev, Z Micro Motion, and X ZCR showed varying degrees of group separation, whereas Y Jerk Mean did not reach statistical significance. This indicates that Y Jerk Mean had limited discriminative value in the present sample. The high-symptom group showed higher values in X Skewness, X Mean, X Std Dev, and Z Micro Motion, suggesting greater waveform asymmetry, higher average acceleration tendency, increased movement variability, and more pronounced micro-movement activity. In contrast, X ZCR was higher in the low-symptom group, indicating more frequent directional switching along the X-axis in this group. Overall, these findings suggest that the difference between symptom groups was not only reflected in general movement magnitude, but also in the organizational structure of movement signals, including axis-specific asymmetry, variability, micro-movement patterns, and zero-crossing characteristics.
(b) illustrates the association between X ZCR and SNAP-IV total scores. The correlation analysis showed a weak negative relationship between these two variables, but the association was not statistically significant (r = −0.17, *p* = 0.444). Visually, the scatterplot suggests a slight downward trend, indicating that children with higher SNAP-IV scores tended to show lower X-axis zero-crossing rates. However, because this relationship did not reach statistical significance, the evidence is insufficient to support a stable linear association between X ZCR and continuous ADHD symptom severity in the current sample. Therefore, X ZCR should be interpreted with caution. Although it may contribute to distinguishing between predefined symptom groups, it cannot yet be regarded as a reliable continuous marker of SNAP-IV symptom scores. This result suggests that X ZCR may be more sensitive to categorical group differences than to gradual changes in symptom severity.
(c) presents the relationship between Z Micro Motion and SNAP-IV total scores. Similar to X ZCR, Z Micro Motion showed a weak negative correlation with SNAP-IV scores, but this association was also not statistically significant (r = −0.20, *p* = 0.361). This result suggests that higher symptom scores may be accompanied by slightly lower Z-axis micro-movement activity, but the observed trend was not strong enough to provide statistical evidence for a stable linear relationship. When considered together with figure a, this finding is informative. Z Micro Motion showed a clearer difference in the between-group comparison, but its correlation with continuous SNAP-IV scores remained weak. This pattern indicates that Z Micro Motion may be useful as a candidate feature for distinguishing symptom-based groups, while its sensitivity to continuous variation in ADHD symptom severity appears limited in the present dataset. Therefore, Z Micro Motion should be interpreted as a potentially group-discriminative movement feature rather than a confirmed continuous symptom indicator.

Taken together, the results suggest group-level differences in selected wearable-derived motor features, but the weak and non-significant correlations with SNAP-IV total scores indicate that these features should not be interpreted as robust continuous markers of ADHD symptom severity.

### 3.4. T–F X-Axis Motor Markers Across Tasks

(a) Group Differences in X-Axis Motor Signatures

X-axis acceleration features were normalized to the Low ADHD group (=100%) to quantify relative alterations in the High ADHD group. Across two motor paradigms, consistent directional alterations were observed in X-axis skewness (T task: 215% vs. 100%, *p* = 0.065) and zero-crossing rate (ZCR) (F task: 114% vs. 100%, *p* = 0.029), respectively. In the T task, elevated X skewness and X mean in the High ADHD group suggested a rightward shift in acceleration distribution. In the F task, elevated X ZCR indicated more frequent sign alternations in the X-axis signal. Notably, X skewness in the F task exhibited the largest effect size but failed to reach significance due to substantial within-group variability.

(b) Representative X-Axis Acceleration Waveforms

Time-domain waveforms of representative High ADHD (H2) and Low ADHD (Z5) participants during the T task (30–90 s) revealed pronounced differences in signal morphology. The High ADHD waveform exhibited larger amplitude and evident rightward skewing, whereas the Low ADHD waveform appeared more symmetric and stable. The overlay of both signals on the same axes facilitated direct visual comparison, with statistical descriptors (μ, σ, skewness, ZCR) annotated for each trace.

(c) X-Axis Time-Frequency Energy Distribution

Time-frequency spectrograms (0–60 s) demonstrated that the High ADHD participant exhibited concentrated low-frequency power (<5 Hz), whereas the Low ADHD participant displayed a more dispersed frequency distribution. This pattern aligned with the skewed waveform morphology in Panel b, collectively suggesting that ADHD-associated motor alterations manifest as atypical spectral composition—specifically, diminished frequency diversity and enhanced low-frequency dominance in X-axis acceleration.

(d) Mann–Whitney U Significance Testing

Mann–Whitney U tests confirmed that the only statistically significant between-group difference was X ZCR in the F task (log_10_(*p*) = 1.53, *p* = 0.029). X skewness in the T task approached but did not reach significance (log_10_(*p*) = 1.19, *p* = 0.065). All other features yielded *p* > 0.10, indicating no reliable group differences. The dual-task design enabled cross-paradigm comparison, revealing that ZCR was most sensitive under structured conditions, whereas skewness exhibited the largest effect size under naturalistic free-living conditions.

The task-wise rank-based comparison in (D) confirmed that X-axis features carried reproducible group-discriminative information. * *p* < 0.05; † *p* < 0.10. Error bars indicate the standard error of the mean. Arrows indicate the direction of the observed association patterns. Taken together, these findings indicate that X-axis motor signatures capture a stable component of ADHD-related movement abnormality across task settings.

**Figure 3:**
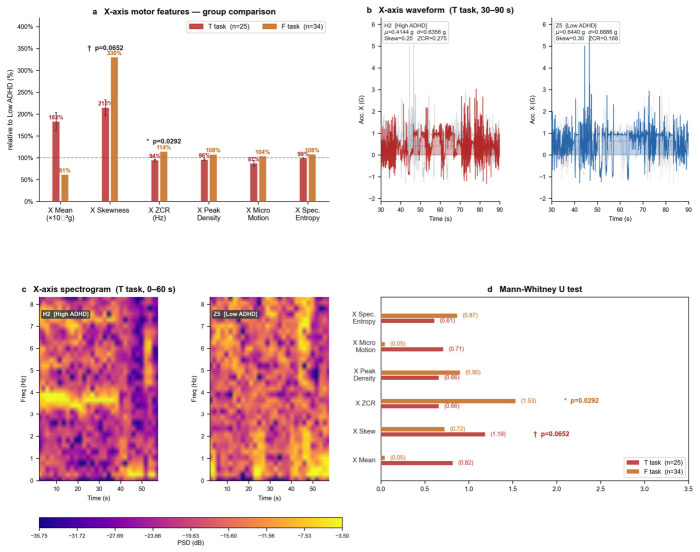
X-axis motor markers across tasks. Note. † *p* < 0.10; * *p* < 0.05.

In the standardized task, X-axis zero-crossing rate (ZCR) was significantly higher in the high-symptom group (*p* = 0.029), indicating that children with ADHD symptoms showed abnormal movement-direction switching during structured task performance. During free activity, X-axis skewness showed the largest effect size; although this effect was only marginally significant (*p* = 0.065), it showed a consistent directional pattern. Time–frequency analysis further revealed concentrated low-frequency energy and a more uniform movement rhythm in children with ADHD symptoms. Together, these findings suggest that X-axis movement alterations, particularly excessive directional switching and distributional skewness, may represent measurable motor features associated with ADHD symptoms in children. (See Figure 3).

### 3.5. Waveform Differences by ADHD Symptom Level

Representative raw accelerometer waveforms are in recording formats. The 1 min recordings were acquired at approximately 100 Hz, whereas the T files corresponded to longer continuous recordings acquired at approximately 30 Hz. Across both formats, the high-ADHD cases displayed a visibly denser oscillatory structure and more frequent short-interval fluctuations than the low-ADHD cases in both the X- and Y-axis signals.

Participant labels (e.g., H2, J34, and W22) represent anonymized participant IDs composed of randomly assigned letter–number combinations and do not correspond to specific clinical characteristics. Red title bars indicate the high-ADHD group, whereas blue title bars indicate the low-ADHD group. Within each subplot, different colored lines represent the X-, Y-, and Z-axis acceleration components.

**Figure 4:**
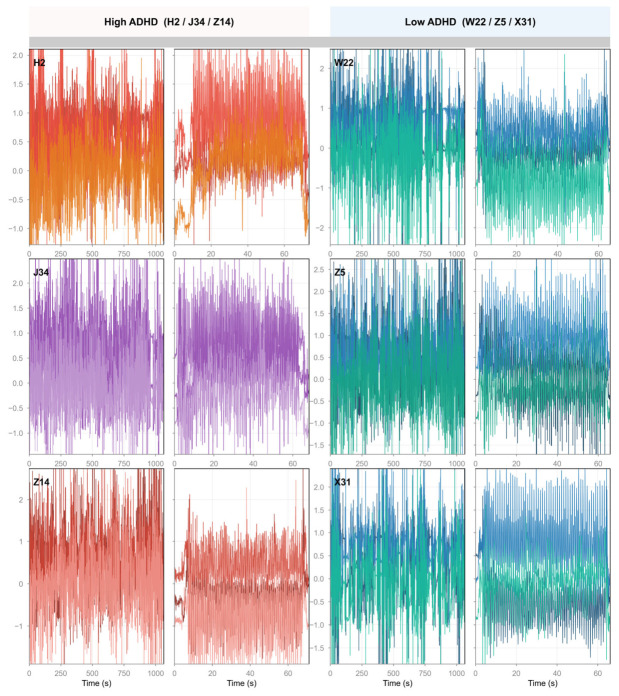
Representative Raw Waveforms.

In the lower-sampling-rate continuous recordings, group differences were most apparent in the waveform’s overall temporal organization. High-ADHD cases showed less stable signal envelopes, more irregular shifts around baseline, and a greater tendency toward rapid alternations without prolonged quiescent segments. By contrast, low-ADHD cases exhibited relatively smoother transitions and more sustained intervals of organized fluctuation. In the 1 min recordings sampled at 100 Hz, these differences became more evident at the local level. The high-ADHD waveforms contained more tightly packed peaks and troughs, shorter inter-oscillation intervals, and a more fragmented appearance. In contrast, the low-ADHD waveforms appeared relatively more regular, with less compressed fluctuation patterns. (See Figure 4).

Importantly, the visual contrast between groups was not limited to gross amplitude. In several panels, the overall amplitude range overlapped substantially between high- and low-ADHD cases. The between-group difference was more consistently reflected in waveform density, local irregularity, and the degree of temporal fragmentation. Taken together, the waveform examples support the quantitative findings by showing that ADHD-related motor abnormalities were expressed primarily as altered signal organization rather than as a simple increase in movement magnitude.

## 4. Discussion

The present study examined whether wearable-derived movement signals could capture motor characteristics associated with ADHD symptoms in children and adolescents. Several findings warrant emphasis. First, children and adolescents with higher symptom burden differed from their lower-symptom peers across multiple accelerometer-derived features, particularly those related to waveform shape, signal variability, directional switching, and local micro-motion. Second, the clearest contrast between groups lay not in overall movement magnitude, but in the internal organization of the signal. Third, the features that best separated symptom-defined groups were not always the same features that tracked symptom severity continuously. Taken together, these findings suggest that group discrimination and symptom-severity tracking may depend on partly different signal properties, a point that is consistent with recent reviews arguing that digital ADHD assessment is increasingly moving beyond simple activity counts toward more structured and interpretable behavioral representations [23].

This interpretation is broadly consistent with earlier work using smartwatches and other objective activity-monitoring approaches in pediatric ADHD. Lin et al. showed that school-aged children with ADHD displayed greater movement variability and more frequent directional switching during daily activities [5], while Lindhiem et al. reported that smartwatch-based sensing could provide an objective estimate of hyperactivity in school-age participants [6]. Related work has further shown that Apple Watch–based systems may be feasible in youth with ADHD [30] and that objective activity monitoring can assist diagnostic discrimination when combined with other performance-based measures [35,36]. The present results are in line with this literature, but they extend it in one important respect: in our data, the most stable differences were reflected less in gross activity level than in fluctuation density, reversals, fragmentation, and cross-axis distributional asymmetry.

This distinction matters because it suggests that ADHD-related motor abnormalities may be more appropriately described as differences in temporal organization than as a simple increase in movement output. In the waveform comparisons, high-ADHD cases tended to show denser oscillatory structure, more frequent short-interval reversals, and a more fragmented signal. This does not necessarily mean that children and adolescents with higher symptom burden simply moved more. Rather, it suggests that their movement may have been organized differently over time. That interpretation is compatible with meta-analytic and review evidence showing that objective movement and sleep abnormalities in ADHD are heterogeneous, context-dependent, and not well summarized by a single global index [37,38], reinforcing the view that temporal structure itself contains clinically relevant information.

Another notable finding is that the features most useful for separating high- and low-ADHD groups were not necessarily the same features that tracked symptom severity continuously. This distinction is important both methodologically and clinically. A variable that maximizes group separation may reflect subgroup membership or threshold-like differences, whereas a variable associated with questionnaire scores across the full sample may capture more graded variation. These two functions are related, but they are not interchangeable. This may help explain why some wearable features appear promising in classification settings yet are less informative for modeling individual differences along a continuum. Recent work in multimodal and machine-learning approaches similarly suggests that prediction improves when multiple signal sources are combined, rather than relying on any single feature family alone [34,39,40]. The present findings are consistent with that pattern and suggest that accelerometer-derived motor features are likely to be most informative when interpreted as one component of a broader behavioral profile.

Several limitations should be considered when interpreting the present findings. First, the sample size was modest, which limits statistical power and may reduce the stability of feature-level estimates. This remains a common limitation in wearable ADHD research, as noted in recent scoping and methodological reviews [37]. Second, the data were obtained from a wrist-worn device. While this offers clear practical advantages, wrist-based signals capture movement at the point of wear rather than whole-body motion directly and may therefore overrepresent distal or context-specific activity patterns [5,6,29,30]. Third, the dataset included multiple recording formats and sampling structures. Although this allowed comparison across conditions, it also introduced methodological heterogeneity. More broadly, this reflects a challenge across the field, where studies continue to differ in devices, preprocessing pipelines, feature construction, and validation strategies [4,37]. Fourth, although sleep-related literature provides a useful context for interpreting movement irregularity in ADHD [39,41,42,43], the present dataset was not designed as a dedicated sleep study; therefore, sleep-related interpretation should remain cautious and secondary to the primary motor findings [44].

An additional point concerns the scope of inference. The present study was cross-sectional and observational. For that reason, the findings should be interpreted as associations rather than evidence of causation. It cannot be concluded from the present data that altered movement organization leads to ADHD symptoms, that ADHD symptoms generate the observed waveform properties, or that one process temporally precedes the other. The results are more appropriately understood as evidence of covariation between wearable-derived movement features and symptom-related measures. This caution is in line with the broader state of the literature, which points to the promise of wearable approaches but also emphasizes the need for longitudinal and intervention-based designs before stronger mechanistic claims can be made [37,38]. In this regard, studies showing that objective signals may change with treatment or contribute to symptom monitoring are encouraging [10,24], but they do not eliminate the need for temporal and developmental validation.

Future research should build on these findings in several ways. Larger samples will be needed to assess the stability of candidate motor features and to determine whether subgroup-discriminative markers remain reliable across age, sex, and symptom profiles. Longitudinal data would help clarify whether the observed movement signatures are developmentally stable, predictive of later outcomes, or sensitive to clinical change [40,45]. It is also likely that multimodal models combining wearable signals with questionnaire data, sleep measures, physiological indices, or treatment information will be more informative than single-source models alone [34,39]. In that sense, the present study is better viewed as a step toward a more interpretable description of wearable-derived motor phenotypes in pediatric ADHD than as a definitive diagnostic framework.

An important limitation of the present study is that participant grouping was based on symptom questionnaire scores rather than formal clinical diagnosis. As a result, the high- and low-symptom groups should not be interpreted as equivalent to clinically diagnosed ADHD and non-ADHD groups. In addition, the dataset did not provide sufficient information on diagnostic criteria, medication status, or the systematic exclusion of psychiatric comorbidities. Therefore, the wearable-derived movement features identified here cannot be interpreted as ADHD-specific motor markers. More cautiously, they should be understood as movement characteristics associated with variation in ADHD-related symptom burden in this sample. Nevertheless, the use of the SNAP-IV remains justified for symptom-based grouping, as the instrument has shown acceptable validity and reliability in previous pediatric ADHD research [32,33]. Future studies using formally diagnosed samples, with clearer control of medication use and comorbid conditions, will be necessary to determine the disorder specificity of these motor features.

Another limitation concerns the incomplete contextual information available for the wearable recordings. Although the dataset documentation distinguished between 1 h aggregated activity data (T files) and shorter activity fragments (F files), it did not provide sufficiently detailed metadata regarding participants’ posture, behavioral state, recording environment, environmental standardization, or dominant-hand placement. Because wrist-derived accelerometer features are highly context-sensitive, this lack of detail limits both the interpretability and reproducibility of the observed feature patterns. At the same time, prior work suggests that the choice of dominant versus non-dominant wrist may not substantially affect activity estimates, which somewhat reduces concern regarding hand-side placement alone [46]. Wearable accelerometry nevertheless remains feasible in pediatric research because wrist-worn devices are unobtrusive and support repeated measurement in naturalistic settings. Accordingly, the present findings should be interpreted cautiously as context-sensitive movement characteristics rather than fully standardized motor markers. Future studies should combine wearable feasibility with better contextual annotation and stricter placement control.

Therefore, the concentration of group differences in X-axis-derived features should not be interpreted as direct evidence that the X-axis is inherently more clinically meaningful than the Y- or Z-axis, nor as definitive evidence of a specific lateral biomechanical abnormality. More cautiously, the current results indicate that symptom-related differences were concentrated in certain device-axis features within this dataset. This interpretation is consistent with broader methodological work showing that accelerometry data are sensitive to choices in data collection, preprocessing, and signal interpretation, and that these factors can complicate the physiological meaning assigned to raw axis-specific signals. Accordingly, the present findings are better understood as device-axis-specific signal differences associated with ADHD symptom variation rather than as fixed biomechanical markers. Future studies should incorporate stricter control of watch orientation, standardized placement procedures, and calibration strategies linking device axes to anatomical movement directions before stronger biomechanical claims are made [47].

More cautiously, the present data indicate that symptom-related variation was concentrated in certain axis- and orientation-derived features within this dataset. This interpretation is consistent with broader evidence linking ADHD to frontal-striatal-cerebellar circuits involved in motor control and executive regulation, while also recognizing that raw accelerometry signals are sensitive to acquisition and processing choices. Because the dataset did not provide full verification of watch orientation or formal axis calibration across participants, some of the observed axis-specific differences may partly reflect device-position factors rather than motor pathology alone. Future studies should incorporate stricter control of watch orientation, standardized placement procedures, and calibration strategies linking device axes to anatomical movement directions before stronger biomechanical claims are made.

This distinction matters because it suggests that ADHD-related motor abnormalities may be better described as differences in temporal organization than as simple increases in movement output. In the waveform comparisons, high-ADHD cases tended to show denser oscillatory patterns, more frequent short-interval reversals, and a more fragmented signal. This does not necessarily mean that children and adolescents with higher symptom burden simply moved more. Rather, it suggests that their movement may have been organized differently over time. This interpretation is broadly compatible with evidence linking ADHD to abnormalities in frontal-striatal-cerebellar circuits involved in motor control, timing, and executive regulation [48]. It is also consistent with neuroimaging evidence showing cerebellar involvement in ADHD, which may be relevant to altered motor coordination and executive-motor integration [49].

A further limitation concerns the biomechanical interpretation of the axis-specific findings. In the present study, the X-, Y-, and Z-axis descriptors were defined in the device coordinate system of a wrist-worn accelerometer rather than in a formally standardized anatomical reference frame. Accordingly, differences concentrated in one device axis should not be interpreted as direct evidence that this axis is inherently more clinically meaningful than the others, nor as definitive proof of a fixed biomechanical abnormality. More cautiously, the present data indicate that symptom-related variation was concentrated in certain axis- and orientation-derived features within this dataset. This caution is also supported by methodological work showing that the analysis and interpretation of wrist-worn accelerometer data remain highly dependent on preprocessing choices and analytic strategy, with no fully standardized consensus across studies [50]. Accordingly, the present findings are better understood as device-axis-specific signal differences associated with ADHD symptom variation rather than as fixed biomechanical markers.

An additional limitation is that the present study did not extend the wearable-derived features into a formal predictive or classification framework. Although the current analyses identified symptom-related differences in motor signal organization, we did not implement or evaluate classification models, receiver operating characteristic analysis, sensitivity, specificity, or cross-validation procedures. As a result, the present findings should not be interpreted as evidence of diagnostic performance or immediate clinical utility. This limits the translational scope of the study, because it remains unclear how well the observed features would distinguish individuals at the case level rather than only at the group level. Future research should therefore examine whether the most informative wearable-derived features retain discriminative value in supervised classification models, and whether such models can achieve stable and clinically meaningful performance under cross-validated and externally validated conditions. In this sense, the current study should be viewed as a step toward feature characterization rather than a predictive biomarker study.

Another limitation is that several developmental and physiological factors that may influence movement patterns were not fully controlled in the present analyses. Age, sex, and body mass index are all known to shape physical activity profiles and movement behavior in children and adolescents, and these effects may introduce variability independent of ADHD symptom burden [51]. In addition, cardiorespiratory fitness may influence free-living activity patterns, self-regulation, and cognitive-motor performance in young people with ADHD, making it a potentially relevant but unmeasured source of heterogeneity in the current dataset [52]. Sleep quality and circadian rhythm are also important considerations, because motor activity in ADHD has been shown to vary across the 24 h cycle, and subgroup differences may partly reflect altered rest–activity regulation rather than symptom-related motor organization alone [53]. This issue is further complicated by medication status, as stimulant treatment may modify circadian motor activity and thereby affect accelerometer-derived features [54]. Relatedly, ADHD subtypes and symptom presentations may differ in their associations with circadian motor patterns, sleep disturbances, and body mass index [55]. Because these variables were not comprehensively modeled in the present study, the reported movement features should be interpreted cautiously as symptom-associated patterns rather than as fully confounder-adjusted markers. Future studies should include these variables as covariates or stratification factors to determine whether the observed feature patterns remain stable after more rigorous developmental and physiological adjustment.

The robustness of the present finding is supported by its convergence with previous wearable-sensor and actigraphic studies of ADHD-related motor activity. In the present study, the X-axis zero-crossing rate showed the clearest and most consistent between-group difference across the T-task and F-task recordings, suggesting that ADHD symptom-related motor differences may involve frequent short-interval directional switching rather than only increased movement magnitude. This interpretation is consistent with smartwatch-based evidence from Lin et al. [5], who used accelerometer and gyroscope data to compare children with ADHD and controls and reported group differences in variance and zero-crossing-rate features. Because zero-crossing rate reflects how often a sensor signal changes direction around zero, both studies point to the relevance of directional-change metrics for characterizing ADHD-related movement organization.

The task-context pattern observed here is also consistent with high-resolution actigraphic work showing that children’s movement features may vary across school contexts and that fine-grained activity features can capture information beyond conventional activity counts (Kam et al.) [56]. More broadly, accelerometer-based methods have been proposed as objective complements to informant-based ADHD ratings because they can quantify aspects of movement timing, variability, and organization that are difficult to capture through parent, teacher, or clinician reports alone (Gawrilow et al.) [57]. Therefore, the current X-axis zero-crossing-rate finding appears methodologically plausible and consistent with prior literature. Nevertheless, because the present study was cross-sectional and symptom-based, the findings should be interpreted as preliminary evidence requiring replication in larger, diagnostically characterized samples.

The results should be interpreted with caution. They support association, not causation, and they underline the need for larger, more standardized, and longitudinal studies before wearable-derived movement features can be regarded as robust clinical markers [23,37].

## 5. Conclusions

The most robust finding of this study is that the X-axis zero-crossing rate consistently differentiated the High ADHD and Low ADHD groups across both T-task and F-task recordings. This indicates that children with higher ADHD symptom levels showed more frequent switching between positive and negative X-axis acceleration directions across different movement contexts. Rather than reflecting a simple increase in overall movement magnitude, the observed pattern suggests that ADHD-related motor differences may be expressed through altered temporal organization of movement, particularly short-interval directional reversals and local movement fragmentation. In contrast, X-axis skewness appeared to capture a complementary aspect of movement organization. Higher skewness values in the High ADHD group suggest a tendency toward waveform asymmetry or directional imbalance, but the statistical evidence for skewness was less consistent than that for zero-crossing rate. Therefore, X-axis skewness should be interpreted as a secondary signal-organization feature rather than the primary finding.

Taken together, these results provide preliminary evidence that wearable-derived X-axis acceleration features can reveal task-stable and task-sensitive components of ADHD-related motor organization. X-axis zero-crossing rate appears to be the clearest cross-task feature, reflecting repeated directional switching, whereas X-axis skewness may reflect context-dependent waveform asymmetry. These findings support a more refined interpretation of ADHD-related motor behavior: the relevant signal is not merely how much children move, but how their movement is temporally structured and directionally organized.

## Figures and Tables

**Table 1:** Demographic and Scale Characteristics of the Study Sample.

Variable	Sex	n	M	SD	Median	Min	Max	Missing n (%)
Height (cm)	Male	30	136.00	10.78	—	120.0	162.0	6 (10.3%)
	Female	22	134.77	10.45	—	120.0	157.0	
Weight (kg)	Male	30	32.27	9.96	—	20.0	59.0	6 (10.3%)
	Female	22	29.82	7.01	—	20.0	42.0	
BMI (kg/m^2^)	Male	30	17.09	3.02	—	11.7	22.8	5 (8.6%)
	Female	23	16.18	2.30	—	12.6	21.8	
SDQ score	Male	30	39.33	13.73	43.00	—	—	
	Female	23	43.52	10.56	44.00	—	—	
SNAP score	Male	30	42.17	19.42	—	—	—	
	Female	22	34.50	12.21	—	—	—	

## Data Availability

The dataset used in the present study was obtained from the publicly available Zenodo repository, “Movement and Mental Health in Children” (DOI: 10.5281/zenodo.14875672). According to the dataset description, the dataset contains wearable movement data collected from children.

## Supplementary Materials

The following supporting information can be downloaded at: https://www.mdpi.com/article/10.3390/bios16060323/s1.

## Abbreviations

The following abbreviations are used in this manuscript:

ADHD	Attention-deficit/hyperactivity disorder
SNAP-IV	Swanson, Nolan, and Pelham Version IV Scale
SDQ	Strengths and Difficulties Questionnaire
ZCR	Zero-crossing rate
IMU	Inertial measurement unit
