# Supplementary material for: Wearable-Derived Axis-Specific Motor Signatures of ADHD Symptoms in Children and Adolescents
Source: Biosensors (Basel). 2026 Jun 2;16(6):323. doi: 10.3390/bios16060323 (PMC13297289; doi:10.3390/bios16060323)
Supplement: Supplementary file 1 [file biosensors-16-00323-s001.zip › biosensors-4302248-supplementary.pdf]

Supplementary Mathematical Definitions and Results Supplement

This supplementary note provides formal mathematical definitions for the principal movement descriptors used in the manuscript and a concise results-oriented interpretation suitable for inclusion in the Results section. All formulas are expressed for a windowed acceleration sequence $a = (a_1, a_2, ..., a_N)$ sampled at $fs = 25$ Hz with window duration $T = N/fs$.

1. Formal Definitions of Core Features

1.1 Fragmentation index (zero-crossing rate, ZCR)

The fragmentation index was operationalized as the zero-crossing rate, that is, the number of sign changes per unit time after centering the signal at its within-window mean. Higher values indicate more frequent directional reversals and a more fragmented, discontinuous movement pattern.

$$\bar{a} = (1/N) \sum_{i=1}^{N} a_i$$

$$ZCR = (1/T) \sum_{i=1}^{N-1} 1[(a_i - \bar{a}) \cdot (a_{i+1} - \bar{a}) < 0]$$

1.2 Micro-motion index (MMI)

The micro-motion index was defined as the proportion of samples whose absolute amplitude fell below a dynamic threshold equal to 10% of the within-window sample standard deviation. Higher values indicate that a larger fraction of the signal is composed of very small-amplitude movement or near-stationary micro-motion.

$$\sigma = \sqrt{ (1/(N-1)) \sum_{i=1}^{N} (a_i - \bar{a})^2 }$$

$$\tau = 0.1 \cdot \sigma$$

$$MMI = (1/N) \sum_{i=1}^{N} 1[|a_i| < \tau]$$

1.3 Oscillatory density (peak density, PD)

Oscillatory density was quantified as the number of local maxima per unit time. A point was treated as a local peak when it exceeded both its immediate predecessor and successor. Higher values indicate more frequent local oscillations and greater rhythm fragmentation.

$$PD = (1/T) \sum_{i=2}^{N-1} 1[a_i > a_{i-1} \wedge a_i > a_{i+1}]$$

1.4 Spectral entropy (Hs)

Spectral entropy was computed from the normalized Welch power spectral density using the Shannon entropy formulation. It quantifies the distributional complexity of spectral energy. Lower values reflect concentration of energy in a narrow frequency range, whereas higher values reflect broader, more irregular spectral organization.

$$N_{seg} = \min(256, N), \quad \Delta f = fs / N_{seg}$$

$$p_k = P(f_k) / (\sum_{j=1}^{K} P(f_j) + \varepsilon), \quad \varepsilon = 10^{-12}$$

$$H_s = - \sum_{k=1}^{K} p_k \log_2(p_k + \varepsilon)$$

In the present implementation, raw Shannon entropy was used directly and was not additionally normalized by $\log_2(K)$.

2. Results-Oriented Supplement

These mathematically defined descriptors were selected because they capture complementary aspects of movement organization that are not reducible to overall movement magnitude. In the context of the present study, ZCR indexed frequent directional reversals and thus served as the primary proxy for movement fragmentation. MMI quantified the proportion of low-amplitude or near-quiescent motion, thereby describing the relative dominance of passive versus active movement states. Peak density summarized the temporal packing of local oscillatory events, and spectral entropy described the degree to which movement-related energy was concentrated or dispersed across frequencies. Taken together, these measures provided a structured description of movement regularity, intermittency, and signal complexity. Their joint use, therefore allowed the Results section to characterize ADHD symptom-related motor differences as differences in temporal organization and signal structure, rather than as a simple increase in gross movement output alone.

In reporting the feature-level results, these descriptors should be interpreted as signal-analysis constructs derived from wrist-worn accelerometry. They support a movement-phenotyping framework in which higher fragmentation, denser oscillatory structure, and greater complexity or asymmetry of device-derived signals are treated as candidate correlates of symptom-related motor variation. At the same time, they should not be overinterpreted as fixed biomechanical abnormalities, particularly when recording context, device orientation, and individual wearing patterns are only partially specified.
